# Supplementary material for: Neuron–Glia Interactions in Tuberous Sclerosis Complex Affect the Synaptic Balance in 2D and Organoid Cultures
Source: Cells. 2021 Jan 12;10(1):134. doi: 10.3390/cells10010134 (PMC7826837; doi:10.3390/cells10010134)
Supplement: Supplementary file 1 [file cells-10-00134-s001.zip › cells-1019787-supplementary/cells-1019787/cells-1019787_original gels.pdf]

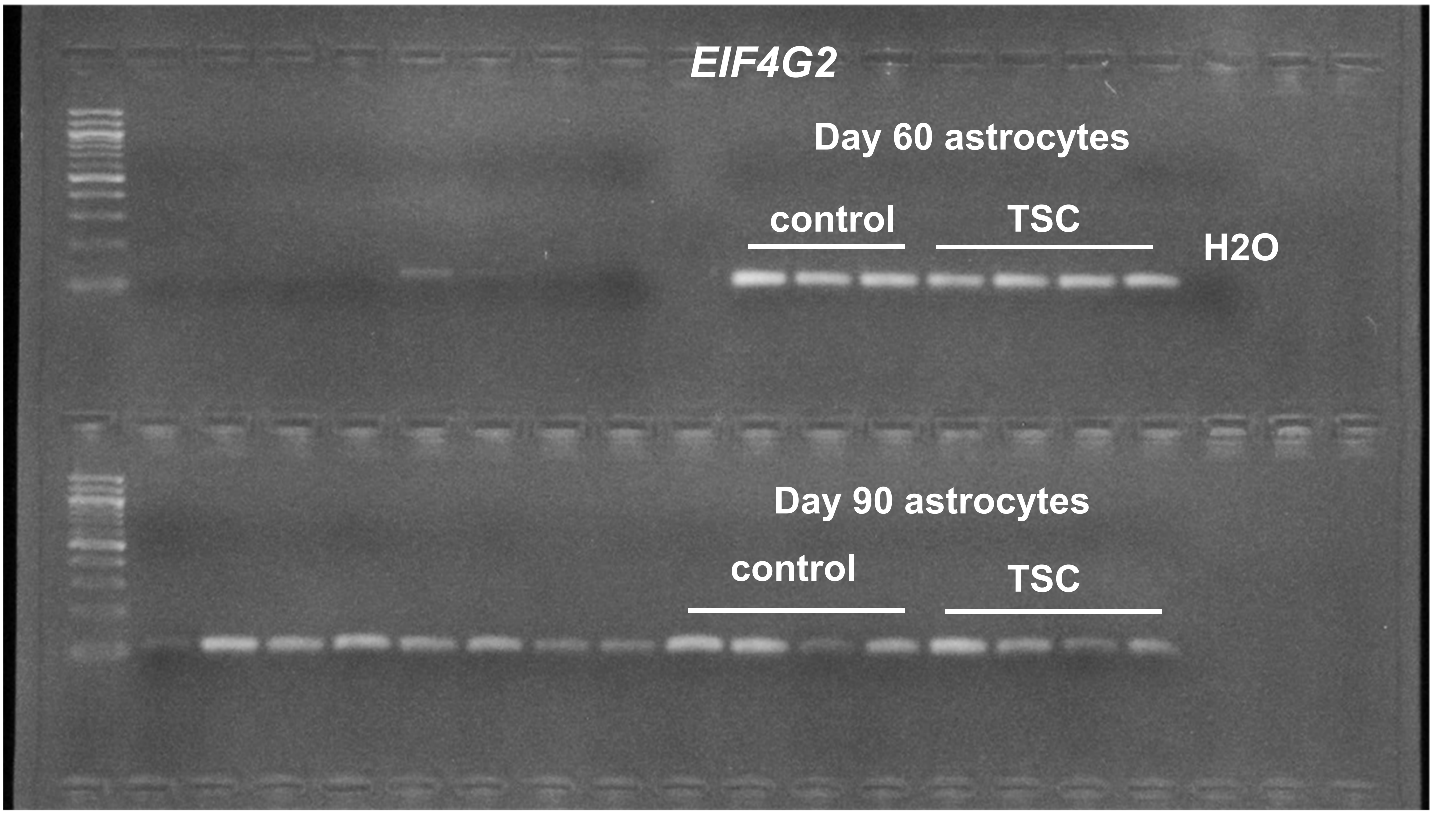
EIF4G2
Day 60 astrocytes
control
TSC
H2O
Day 90 astrocytes
control
TSC

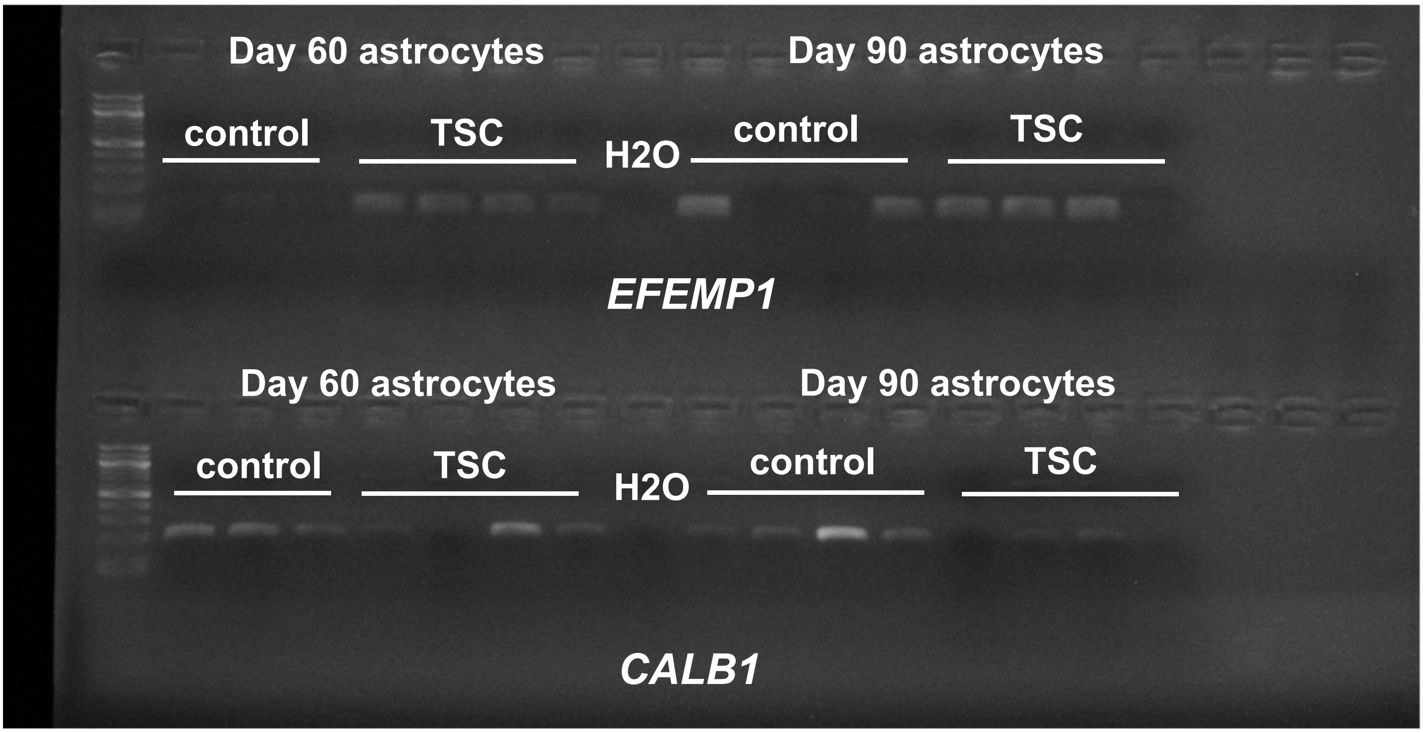
Day 60 astrocytes
Day 90 astrocytes
control
TSC
H2O
control
TSC
EFEMP1
Day 60 astrocytes
Day 90 astrocytes
control
TSC
H2O
control
TSC
CALB1

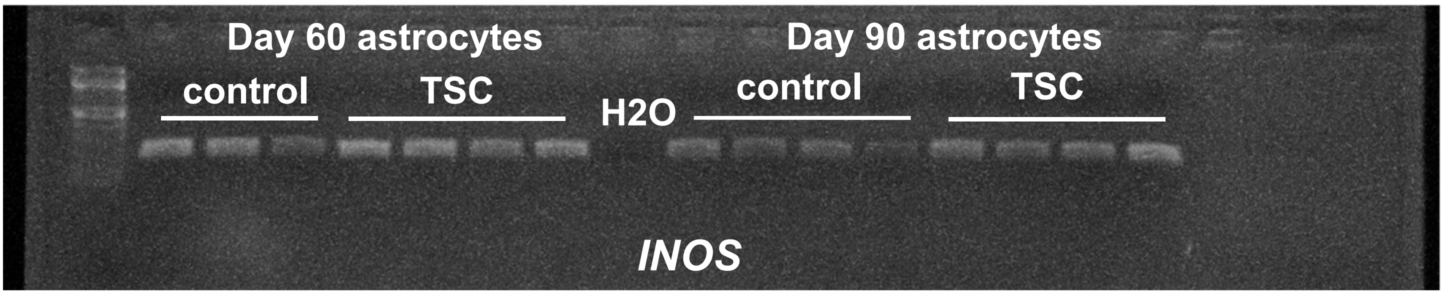
Day 60 astrocytes
Day 90 astrocytes
control
TSC
H2O
control
TSC
INOS

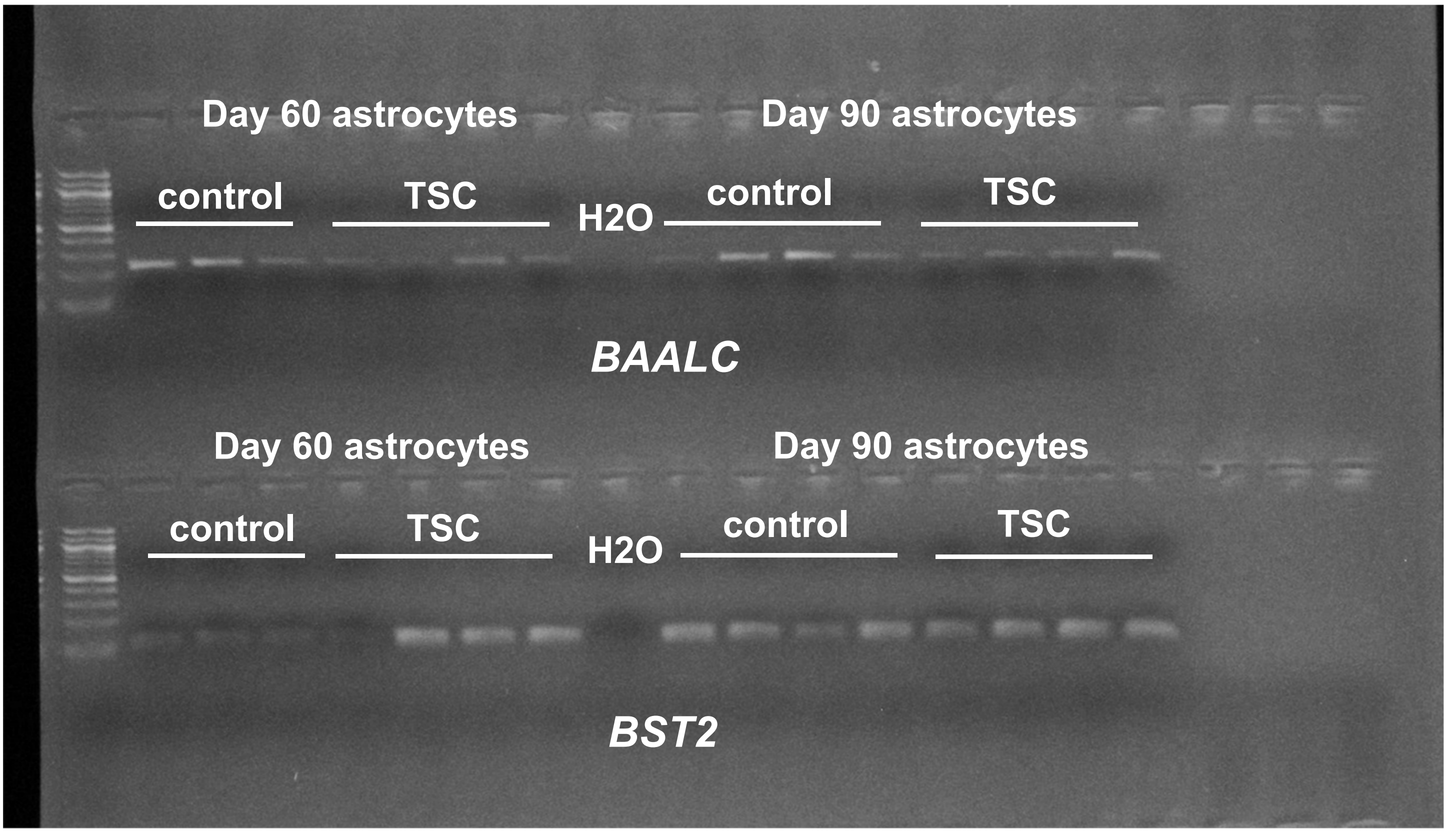
Day 60 astrocytes
Day 90 astrocytes
control
TSC
H2O
control
TSC
BAALC
Day 60 astrocytes
Day 90 astrocytes
control
TSC
H2O
control
TSC
BST2

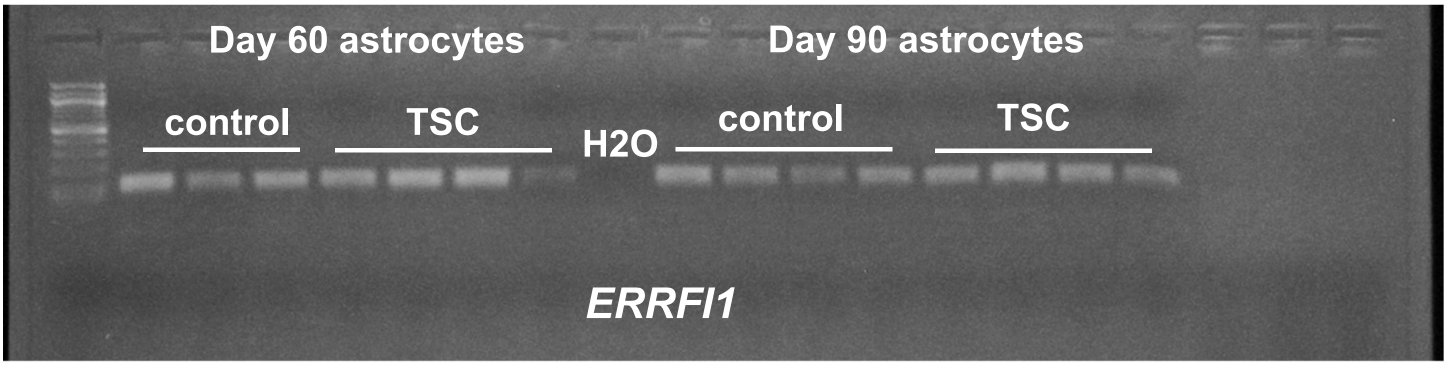
Day 60 astrocytes
Day 90 astrocytes
control
TSC
H2O
control
TSC
ERRFI1

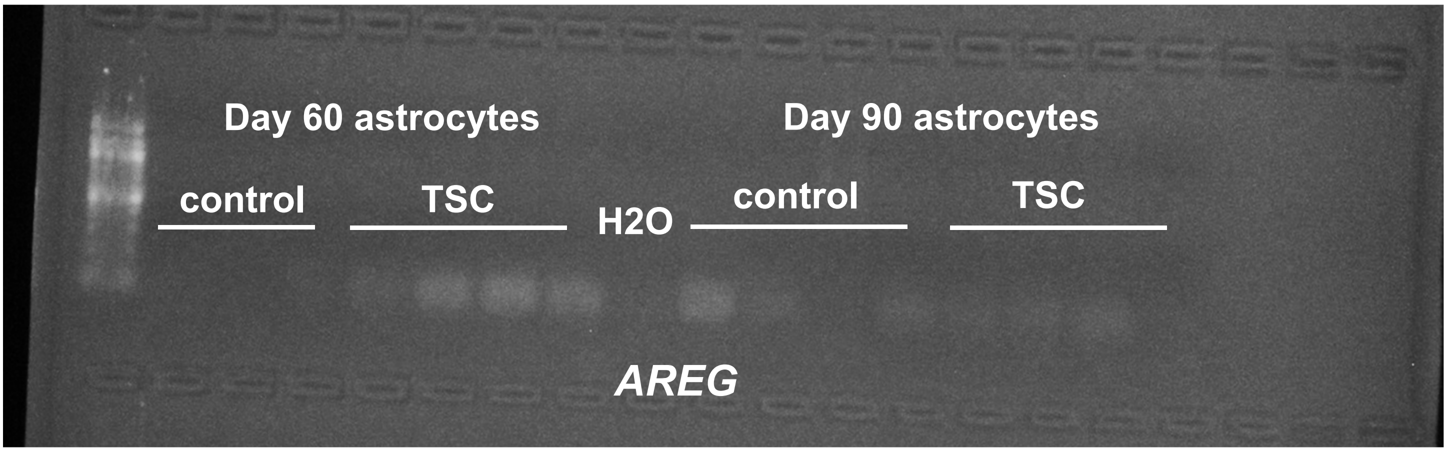
Day 60 astrocytes
Day 90 astrocytes
control
TSC
H2O
control
TSC
AREG
