# Supplementary figures and images for: Neuron–Glia Interactions in Tuberous Sclerosis Complex Affect the Synaptic Balance in 2D and Organoid Cultures
Source: Cells. 2021 Jan 12;10(1):134. doi: 10.3390/cells10010134 (PMC7826837; doi:10.3390/cells10010134)

Figure S1. Organoids of the same iPSC line show stable mRNA expression patterns

A

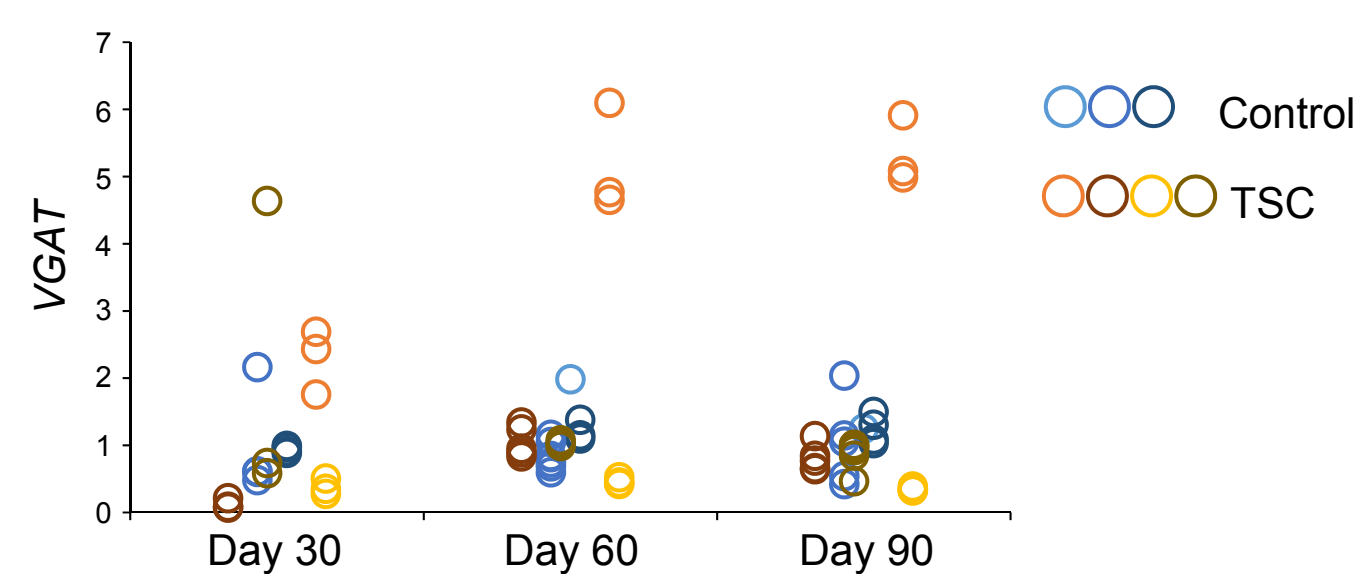

B

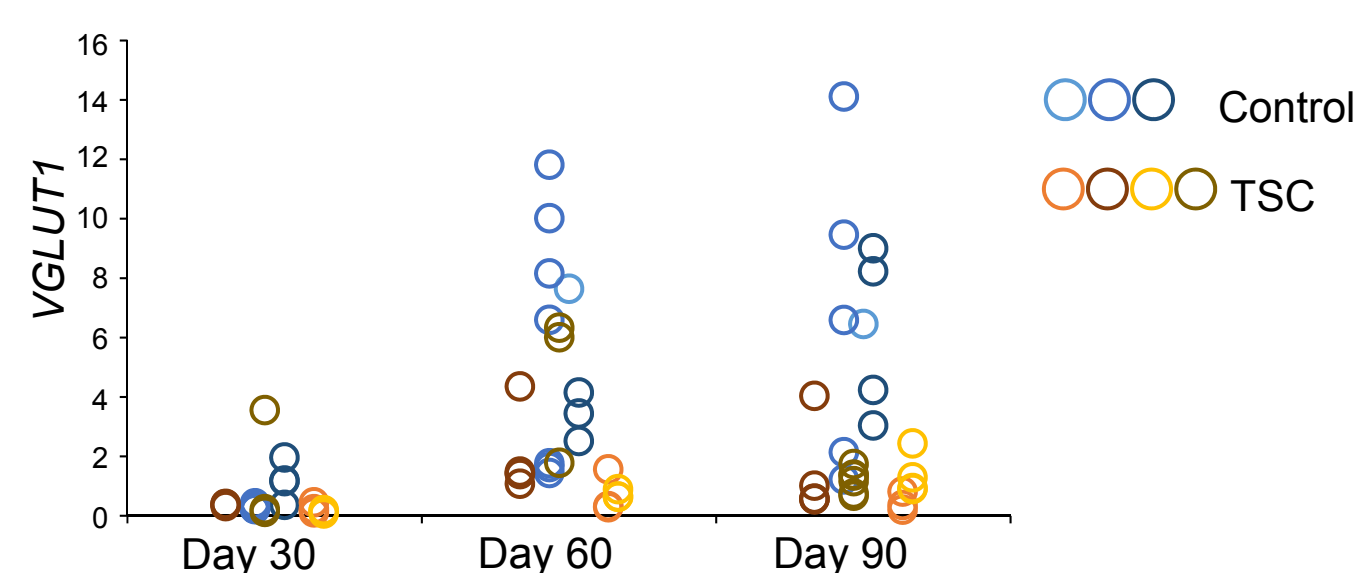

Supplement: Supplementary file 1 [file cells-10-00134-s001.zip › cells-1019787-supplementary/cells-1019787/cells-1019787 FigureS1.pdf]
